# Specific Aspects of Breast Cancer Therapy of Elderly Women

**DOI:** 10.1155/2016/1381695

**Published:** 2016-10-11

**Authors:** Petra Tesarova

**Affiliations:** Department of Oncology, 1st Faculty of Medicine, Charles University in Prague and General University Hospital, Prague, Czech Republic

## Abstract

Breast cancer is the leading cause of death among women, and its incidence increases with age. The average age at diagnosis is 61 years, and the majority of deaths occurs after the age of 65 years. Optimal approach to elderly women with breast cancer is still a major challenge. Elderly patients with cancer should have at least a brief geriatric assessment to detect potentially treatable problems not always adequately evaluated by the oncologists. Therapeutic nihilism should be avoided and effective treatment provided, unless there are compelling reasons against it. Sharing the care for the patient with geriatricians or primary care physicians trained in geriatrics should be considered for all vulnerable and frail elderly patients.

## 1. Introduction

Increasing age is the primary risk factor for the breast cancer. The median age for breast cancer at diagnosis is around 60 years, and over 40% of women with newly diagnosed breast cancer are aged 65 years or older [1]. As by the year 2030 twenty percent of the population is anticipated to be aged over 65 years we can expect that in the near future the proportion of elderly women with early breast cancer will probably grow considerably.

Higher noncompliance with treatment, presence of comorbidities that can contribute to mortality, and the high cost of the treatment are among the factors that explain why most of the screening strategies for breast cancer do not include patients over 70 years of age. Elderly patients with breast cancer (older than 70 years) are usually excluded from current screening strategies. The consequence is that the diagnosis of breast cancer in the elderly patients is often made late and about 48% of patients older than 65 years of age have metastases already at the time of diagnosis [2].

There are few guidelines for the management of elderly patients with breast cancer, primarily due to the lack of evidence and the lack of representation of elderly women in randomized controlled trials testing the efficacy and safety of adjuvant therapy, and the oncologists must often make treatment decisions in this area of uncertainty. This may frequently lead to undertreatment or, less commonly, overtreatment of the patient and consequently may result in poorer outcomes [3].

Since 1990, breast cancer death rate has been steadily decreasing [4]; this improvement, however, has preferentially been documented in women younger than 75 years old. Comparison between breast cancer death rates in 1990 and 2007 demonstrated that while the mortality rate due to the breast cancer in the general population decreased by 2.5% per year in women aged <75 years, mortality rate due to breast cancer decreased by only 1.1% per year in women aged ≥75 years [5]. In Europe mortality due to breast cancer decreased between the years 1990–1994 and 2000–2004 by 13%; however, the decrease was much more pronounced in women aged 35–64 years at 17%, compared with only 6% for women aged ≥65 years [6].

## 2. Elderly Women

Chronological age of at least 65 years defines elderly (or older) persons in most developed countries, but the definition may not be easily applicable to many developing countries. Life expectancy by nation at birth in year 2011 ranged from 48 to 82 years between developing and developed countries. Women used to have a lower mortality rate at each age [7].

Common use of the calendar age to mark the threshold of old age assumes the equivalence with the biological age, yet, at the same time, it is generally accepted that these two are not necessarily identical and biological age is also influenced by the socioeconomic status [8].

Most people in the age range of 60–80 years may enjoy high-quality life, but the condition of frailty characterized by “bodily failure” and greater dependance becomes increasingly more common with increasing age. A group of geriatricians proposed a general definition of frailty as “a physical state of increased vulnerability to stressors that results from decreased reserves and dysregulation in multiple physiological systems” [9].

## 3. Screening

The major determinants of the improvement of the survival in breast cancer appear to be breast cancer screening and adjuvant therapy [5]. However, regarding the screening, the US Preventive Services Task Force stated that for women ≥70 years there are insufficient data to determine the effect of mammographic screening on breast cancer mortality [10]. In the Czech Republic there is no upper age limit for the screening, but the data on the age-specific impact of the breast cancer screening on the outcome of the patients are not yet available [11]. Randomized trials studying advantages of screening mammography have excluded women aged above 74 years. Thus, the benefits of screening in this subgroup of the population are unknown. Observational studies, however, suggest that elderly women with a life expectancy of more than 10 years should be included in mammography-based screening policies. These studies estimate that the mortality is reduced by 0.2% when women aged above 70 years undergo screening mammographies on biannual basis for 10 years rather than discontinuing this test at the age of 69 years. In a review by Marmot et al. [12], the panel encouraged breast cancer screening in the UK because it reduced breast cancer mortality by 20% in 10,000 UK women aged 50–70 years invited for screening mammography every 3 years. About 681 cancers were diagnosed and 43 deaths were prevented, but there were also 129 false positive findings. The screening definitely has benefits such as reducing breast cancer mortality. Some studies, however, stated that the benefit is minimal against a nonnegligible overdiagnosis, which presents a source of anxiety for women [13]. The choice between the benefits and harms of screening should be shared with women who agreed to be screened.

## 4. Factors Affecting the Choice of Therapy

Breast cancer in the elderly is thought to have a good outcome, compared with breast cancer in younger patients [14], but the prognosis varies considerably depending on many factors; older patients might therefore be at risk of being over- or undertreated, because any of these factors can alter the risk-benefit balance in the consideration of breast cancer treatment [15].

## 5. Tumor Biology

In most studies, the prevalence of tumors with more indolent features is higher in elderly compared with younger women [16]. As an example, in a combined series of women aged 55 years or older with breast cancer derived from the San Antonio Breast Cancer Database (*n* = 35,154) and the Surveillance, Epidemiology, and End Results (SEER) registry (*n* = 171,424), breast cancer diagnosed in elderly women had lower proliferative indices, normal p53 expression, and diploid DNA, compared with breast cancer diagnosed in younger women [17].

Breast cancer in elderly women expresses estrogen (ER) and/or progesterone (PR) receptors more often than in younger women (85 versus 70% in women ≥65 years versus <50 years, resp. [8]). Amplification and/or overexpression of the human epidermal growth factor 2 receptor (HER2-positivity) is less commonly seen in elderly women; in one series, the proportion of cases that were HER2-positive was 4% versus 9% percent among women over 60 years compared with those ≤35 years. However, similarly as in younger women overexpression of HER2 is associated with a poor outcome [18].

Age does not affect significantly histologic features of breast cancer. However, lobular, mucinous, papillary carcinomas are slightly more common in elderly women. Mucinous carcinomas represent between four and six percent of breast cancers in women older than 75 years; in contrast, they represent only one percent of cases in premenopausal women. These are usually small, slowly proliferating, and low-grade tumors.

## 6. Comorbidity and Performance Status

Biological age, comorbidity, and functional status are important factors to be considered in treatment decisions in elderly cancer patients. In general, advancing age is associated with reduced tolerance to physiologic stress, higher prevalence of comorbidity, reduced social support, cognitive impairment, and frailty. Comorbidities increase with age and cancer patients in their 70s may be expected to suffer on average three comorbid conditions [19]. Comorbidities, such as renal failure, liver disease, and/or cerebrovascular disease, have been associated with an increased risk of death from causes other than breast cancer, independent of age [20]. The presence of comorbidity is independently associated with decreased life expectancy and plays a major role in determining survival in elderly patients with cancer [21, 22]. In a large observational cohort study from Denmark, the presence of comorbidities, as measured by the Charlson Comorbidity Index, was demonstrated to be an independent adverse prognostic factor in patients with breast cancer aged 50–79 years [22]. Chronological age itself, therefore, is not an appropriate criterion on which to decide appropriateness of adjuvant therapy. Instead, biological age, which refers to the presence of comorbidities and the general fitness or health of a patient, should be considered.

The impact of a breast cancer diagnosis on life expectancy was addressed in a study that used the linked SEER-Medicare dataset to compare the survival of 66,000 women older than 67 years of age who were diagnosed with breast cancer with control subjects without breast cancer matched for age, comorbidity, prior mammography, and social demographics [23]. Women diagnosed with ductal carcinoma in situ (DCIS) or stage I invasive breast cancer had a lower risk of death compared with controls (adjusted hazard ratio [HR] 0.7, 95% CI 0.7-0.7 for DCIS; HR 0.8, 95% CI 0.8-0.8 for stage I breast cancer). Cardiovascular disease was the most common cause of death in these women. Women diagnosed with stage II disease or higher had an increased risk of death compared with controls (HR 1.2, 95% CI 1.2-1.2), regardless of age. However, among women ≥80 years diagnosed with stage II breast cancer, cardiovascular disease was still the most common cause of death. For women with stage III or IV breast cancer, breast cancer was, however, the most common cause of death even in these very elderly patients.

All elderly women should undergo a pretreatment evaluation including an assessment of organ function and comorbidity, which may have a decisive impact on the ability of patients to tolerate surgery or anticancer therapies and, in particular, chemotherapy [24].

Functional status refers to one's ability to perform routine daily tasks. The impact of functional status was demonstrated in the study of 2202 women with breast cancer who had completed adjuvant treatment and provided information on endurance, muscular range of motion, and dexterity. The presence of functional limitations was significantly associated with older age, less education, and obesity. With a median follow-up of nine years, patients with functional limitations had an increased risk of death from all causes (HR 1.40, 95% CI 1.03–1.92), but not from breast cancer (HR 0.90, 95% CI 0.64–1.26) [25].

Ideally, elderly patients with a diagnosis of early breast cancer should undergo full geriatric assessment or geriatrician review, as this can not only more accurately determine a patient's biological age, but also detect functional deficits that may be missed on routine oncological review. Despite the importance of adequate geriatric assessment, oncologists refer their patients to geriatricians quite infrequently [26]. Among elderly patients with breast cancer, frailty is associated with an increased risk of treatment-related complications, including a higher likelihood of requiring hospitalization, and decreased overall survival. Frailty is characterized by decreased reserve and diminished resistance to stressors, which results from cumulative declines across multiple physiologic systems and can lead to increased susceptibility to adverse outcomes [27]. A reasonable definition of frailty comes from the Cardiovascular Health Study index, which identified frailty criteria in a longitudinal study of over 750 adults aged 70 and older: weight loss ≥5 percent in the last year, slow walking speed, and decreased physical activity [28].

## 7. What Is the Optimal Treatment for Elderly Patients?

Elderly women generally did not participate in clinical trials evaluating the treatment of breast cancer, as the eligibility criteria usually excluded most elderly patients for different reasons, namely, the presence of comorbidities. As a consequence there is a lack of evidence-based guidelines dedicated to the treatment of breast cancer in this population. Variations in treatment patterns among elderly and younger women may be due to differences in breast cancer features, presence of competing comorbidities, and the general health status of women. Age-related changes in pharmacokinetics and metabolism are rare [29].

## 8. Impact of Age on Treatment

Following a new diagnosis of breast cancer, elderly women often receive less than standard therapy. In the largest study, which involved over 120,000 women, increasing age was associated with the decreased surgical rates. While over 93% of women <80 years underwent surgery, the surgical rates were 83%, 65%, and 41% for women aged 80 to 84 years, 85 to 89 years, and ≥90 years, respectively. There was also less frequent use of adjuvant radiation therapy (RT) following breast conservation surgery; RT was administered in >90%, 86%, 71%, 36%, and 15% of women aged <75 years, 75 to 79, 80 to 84, 85 to 89, and ≥90 years. However, whether the use of RT varied according to the prognostic factors of the tumor (e.g., tumor size, hormone receptor status) was not reported. On the other hand, primary endocrine therapy (without surgical treatment) was administered in a higher proportion of women as age increased. This ranged from <1% among women aged less than 65 years to 47% among women 90 years or older [30].

Whether these factors and/or patient choice influence which treatments are administered is also not entirely clear. As an example, one prospective study included 800 women (aged 70 and older) with operable (stages I to IIIA) breast cancer to determine the impact of age on breast cancer surgery. In multivariate analysis women 85 years and older had significantly lower odds of being treated for breast cancer by surgery even after adjustment for the patient choice and functional health status (odds ratio [OR] 0.18, 95% CI 0.07–0.44). These data suggest that while health measures may help to explain the lack of surgery in most elderly women, they do not necessarily explain the lack of surgical treatment in women 85 years and older [31].

## 9. Adjuvant Treatment

For otherwise healthy elderly women recommended treatment according to standard guidelines for breast cancer could be used. In general, healthy elderly women tolerate breast cancer treatment as well as younger patients and are not at increased risk of adverse events [32]. For medically frail patients (e.g., those with cognitive impairment, frailty, and/or comorbidities) treatment depends on whether or not surgery is an option. In patients who are surgical candidates surgical resection of the primary tumor is preferred to the medical therapy. In most cases these patients can be only observed after surgery and may not require any further therapy. For patients who refuse surgery and those patients who are not surgical candidates primary medical therapy is offered based on the primary tumor features. However, for women with a limited life expectancy (due to comorbidities) and those who wish to avoid treatment-related toxicity, supportive care and referral for palliative care services may be the recommended option.

## 10. Comprehensive Geriatric Assessment

For women over 65 to 70 years diagnosed with breast cancer, we perform a clinical risk assessment using standard clinical and laboratory assessments. In addition, we advocate a two-step approach using a brief screening tool for geriatric syndromes in all patients. Based on the results of the screening, a comprehensive geriatric assessment (CGA) may help physicians to develop a coordinated plan of the treatment of breast cancer.

Comprehensive geriatric assessment (CGA), which takes approximately 45 min to perform, is designed to capture details regarding the physical, nutritional, and psychological functioning of the elderly patient and can help to more accurately determine the biological age of the patient. Detection of different aspects of the functional deficit may then guide the management of reversible deficits, for example, malnutrition, and thus improve the tolerability of treatment, quality of life, and survival. CGA adds further information on the patient fitness over that of performance status and has been shown to be an independent predictor of survival irrespective of tumor type or performance status [33].

CGA can identify elderly cancer patients who are potentially fit enough for adjuvant chemotherapy and who otherwise would not be treated based on chronological age. The International Society of Geriatric Oncology recommends incorporation of CGA, with or without screening, for all elderly cancer patients and, moreover, suggests the use of serial geriatric assessments, to identify as early as possible incident deterioration, for which specific interventions can then be targeted [34]. Some experts prefer the use of a screening tool (assessment of autonomy, malnutrition, depression, cognition, and comorbidity) to identify vulnerable patients for whom the CGA could potentially optimize their treatment of cancer [35].

Studies have been also performed to identify shorter evaluation tools that have good correlation to the full CGA (aCGA) in order to better stratify patients as being sufficiently fit enough to begin breast cancer treatment immediately and those who are not.

A major advantage of aCGA is its brevity and the relative simplicity, such that over 85% of patients and 100% of physicians might be able to complete their sections without difficulty or assistance. aCGA also meets the feasibility end points set for consideration of its use in future CALGB trials in elderly cancer patients [36].

Other tests were used for the evaluation of the frailty in elderly patients with cancer, for example, Vulnerable Elders Survey 13 (VES-13), Cardiovascular Health Survey (CHS), Triage Screening Tool (TRST 1+), Groningen Frailty Index, or Geriatric 8 (G8) frailty screening tool, but they were shown to have especially lower specificity compared to CGA and cannot reliably identify the patients who should then undergo the complete CGA [37–40].

However, while CGA can identify and allow the management of reversible deficits in geriatric domains, which can translate into improvements in treatment compliance, tolerability, quality of life, and survival, there is limited evidence that geriatric assessment with CGA actually impacts on treatment decision-making in the oncology setting [41]. We would need best of all the screening which would identify the best (fit) and the worst (frail) patients immediately, on whom treatment decisions could be readily made. Healthy elderly (fit) patients could proceed directly to anticancer treatment; frail patients could proceed to palliative one.

In fact, shorter evaluation test with good correlation with the full CGA could give us the chance to quickly stratify the patients into those being fit (healthy) enough to begin breast cancer treatment immediately (without the need to perform the complete CGA) and those unfit (frail) who should either undergo complete CGA or be indicated directly to the palliative care only. Until these tests are validated CGA should be performed in all patients with the additional advantage of the identification of potentially reversible (correctable) problems.

## 11. Healthy Elderly Patients

The treatment approach to healthy elderly women with newly diagnosed nonmetastatic breast cancer is identical to that of younger women and should include surgery for removal of the cancer from the breast, axillary assessment (if indicated), radiation therapy (if indicated by the type of surgery and cancer size and stage), and systemic adjuvant treatment (depending on the tumor characteristics and recurrence risk). Most elderly women are likely to choose breast conservation surgery over mastectomy [42]. Neoadjuvant systemic therapy can be offered to some patients, especially if they are interested in breast conserving therapy [43]. The surgical options for the axillar involvement are identical to those offered to younger women. The risk of the local recurrence is lower in older women and the benefits of RT following breast conservation surgery may decline with age [44]. Therefore, some elderly women may not require adjuvant RT, particularly those who are older than 70 years with small (<2 cm) estrogen receptor-positive breast cancer and no evidence of nodal disease (either clinically or pathologically confirmed), and agree to take adjuvant endocrine therapy. Patients who prefer not to proceed with adjuvant RT should be counselled that they may have a slightly higher risk of the in-breast cancer recurrence compared with those who undergo RT.

The same principles for the use of adjuvant systemic therapy in younger individuals also apply to healthy elderly women. In general, administration of the anthracycline- and/or taxane-based regimen is preferred treatment in healthy elderly women. However, the benefit of the treatment must be balanced against the risks of anthracycline-based therapy, especially the heart damage [45]. This was shown in a study using Surveillance, Epidemiology, and End Results- (SEER-) Medicare database that included 43,338 women aged 66 to 80 with stage I to III breast cancer with no history of preexisting heart failure. Over 4700 women received an anthracycline-containing chemotherapy regimen, while almost 4000 received a non-anthracycline-containing regimen; 34,705 women received no chemotherapy. At a median follow-up of 56 months, the subsequent heart failure rates were reported at 5 and 10 years. For women treated with anthracyclines they were 19% and 38%, respectively, in women treated without an anthracycline 18% and 33%, respectively, and in women not treated with chemotherapy at all 15% and 29%, respectively. Besides age, other baseline characteristics associated with the development of heart failure included black race, hypertension, diabetes, and coronary artery disease. Epirubicin may be less cardiotoxic than doxorubicin. It is always necessary to follow a cumulative dose of anthracyclines and carry out monitoring of cardiac function using MUGA scans or echocardiography.

In patients who are not candidates for an anthracycline we administer docetaxel with cyclophosphamide (TC). This approach is based on one randomized controlled trial which demonstrated that TC results in a higher disease-free and overall survival compared to doxorubicin plus cyclophosphamide (AC) in women with stage I to III breast cancer (16% of whom were 65 years or older) [46]. Another possibility may be the CMF therapy, although it may be considerably haematotoxic, especially in elderly women, and then weekly paclitaxel [47].

Capecitabine monotherapy is not recommended as an adjuvant chemotherapy regimen in elderly women with early-stage breast cancer. In the Cancer and Leukemia Group B 49907 trial, women aged 65 years or older were treated with AC or capecitabine, per clinical choice [48]. Based upon analysis of 633 women enrolled and followed up for a median duration of 2.4 years, capecitabine resulted compared to AC in the lower rate of relapse-free survival (68% versus 85%) and overall survival (86% versus 91%) at three years.

The Cancer and Aging Research Group (CARG) performed a prospective observational study that included 500 elderly patients with cancer (not limited to breast cancer) to determine which clinical factors predicted serious (grade 3 to 5) chemotherapy-related toxicities. Factors significantly associated with increased toxicity risk included age > 71, primary cancer of the gastrointestinal or genitourinary tract, administration of full dose-chemotherapy without dose reduction, use of multiagent chemotherapy regimen, anemia, low creatinine clearance, worse hearing, more than one fall in the last six months, inability to independently take medications, limited ambulatory ability, and decreased social activity due to physical or emotional limitations [24].

In a separate study, 562 elderly cancer patients were prospectively followed up to define the Chemotherapy Risk Assessment Scale for High-Age Patients (CRASH) score [49]. Predictive outcomes associated with either the CARG or CRASH score indicated that the risk prediction for short-term toxicity may require the evaluation of functional information and nutritional status in addition to the well accustomed clinical and laboratory parameters for treatment decision-making. In keeping with that, the European Organisation for Research and Treatment of Cancer (EORTC) group decided on a minimum dataset (minDS) as a baseline assessment and screening of elderly cancer patients in their future trials. The minDS includes the Charlson Comorbidity Index, Geriatric 8 (G8), Instrumental Activities of Daily Living (IADL) questionnaire, and one social question regarding living situation [50].

For healthy elderly women with HER2-overexpressing breast cancer who have undergone surgery, adjuvant trastuzumab plus systemic therapy is recommended rather than adjuvant systemic therapy alone. Combined treatment significantly improves survival and diminishes recurrence risk. As is the case in younger women in whom trastuzumab is recommended, one year of adjuvant trastuzumab should be administered to the elderly patient, and most elderly patients are able to complete this treatment without complications. Note should be made that the use of nonstandard chemotherapy regimens with trastuzumab may be less toxic, but they may not provide the same survival benefit as standard trastuzumab-containing regimens [51]. The administration of HER2-directed therapy plus chemotherapy is associated with a small, but real increase in the risk of myocardial dysfunction, particularly if an anthracycline-based regimen is administered. Therefore, the risk associated with combined treatment must be balanced against the prognosis of women with newly diagnosed breast cancer [52].

Adjuvant endocrine therapy should be offered to all women with ER-positive breast tumors, regardless of age, provided they are candidates for medical therapy. In elderly women we prefer to administer an aromatase inhibitor (AI) because of its benefits in the adjuvant setting compared with tamoxifen. However, for women at risk of cardiovascular complications, or bone loss, and those unable to tolerate an AI due to its toxicity, tamoxifen alone is a reasonable alternative [53]. As in younger women, the optimal duration of the use of endocrine therapy is not clear. A minimum duration of five years of endocrine therapy should be prescribed for most elderly women. However, longer durations up to 10 years might be appropriate in selected patients, especially those with tumors with higher risk features (e.g., nodal involvement, or higher tumor grade [54]).

## 12. Frail Elderly Patients

For frail* elderly* patients (e.g., those with cognitive impairment or comorbidities), the risks of surgery, radiation therapy, chemotherapy, and endocrine therapy must be considered in the development of an individualized treatment plan. Women with a limited life expectancy and those who wish to avoid treatment-related toxicity should be offered supportive care and referral for palliative care service. For patients who are surgical candidates surgery is preferred to the primary endocrine therapy for women with hormone receptor-positive breast cancer because surgical resection of the breast cancer reduces the risk of the local recurrence which can be a source of significant morbidity in elderly women. However, the impact of surgery on overall survival in elderly women has not been clearly demonstrated. Multiple studies of women with hormone receptor-positive breast cancer treated with either surgery alone or surgery followed by endocrine therapy consistently showed improvements in the risks of recurrence compared to endocrine therapy alone [55]. Following surgery, most patients can be offered observation only, especially if their life expectancy is limited. For patients who desire subsequent treatment, adjuvant therapy can be administered using an approach similar to that used for medically frail patients who did not undergo surgery.

For frail elderly patients with breast cancer who are not candidates for surgery, including those who refuse surgery and desire treatment for breast cancer, systemic therapy based on the primary tumor features may be offered. However, for women with a limited life expectancy (due to comorbidities) and those who wish to avoid treatment and its associated toxicity, we proceed with supportive care and referral for palliative care services.

There are no randomized controlled trials comparing endocrine therapy to more aggressive treatment (i.e., chemotherapy) or to observation only in women who did not undergo primary surgery for breast cancer, nor are there prospective studies evaluating tamoxifen versus an AI in this population. However, based on the incremental benefits of an AI on survival outcomes compared to tamoxifen when administered in the adjuvant setting for women with postmenopausal breast cancer, an AI is preferred. Tamoxifen is a reasonable alternative in women who do not tolerate an AI [56]. We administer radiation therapy (RT) as the sole treatment of breast cancer very rarely, only in medically frail patients. RT might be considered if comorbidity or frailty precludes surgery, the tumor is not responsive to endocrine therapy, and local control is necessary for palliation. The available data suggest that long-term results following primary RT are inferior to surgery [57].

## Additional Points

*Summary*. Breast cancer is a major health problem in the geriatric population. However, elderly women often are treated less aggressively than younger women, even after adjustment for confounding factors, such as comorbidity, social support, and functional status.

Patients with breast cancer over age 65 to 70 years should be screened for geriatric syndromes using a brief tool. Based on the results of the screening, a comprehensive geriatric assessment (CGA) may help physicians to develop a coordinated plan for breast cancer treatment. In our opinion, the benefits and harms of screening should be discussed with the patient.

The treatment approach to healthy elderly women with newly diagnosed nonmetastatic breast cancer is similar to that of younger women. The treatment plan consists of the surgery of the breast and axilla, radiation therapy if applicable, and systemic treatment where indicated per standard guidelines.

We suggest to consider observation rather than adjuvant treatment following surgery, if life expectancy of the patients is limited due to comorbidities not directly related to the breast cancer.

In medically frail elderly women with breast cancer and positive hormone receptors who are not candidates for surgery (including those who decline surgery) and who desire treatment despite the risk of toxicity associated with systemic therapy primary endocrine therapy rather than chemotherapy can be offered. In patients with hormone receptor-negative disease, we suggest single agent chemotherapy rather than combination chemotherapy. In patients with HER2-positive disease (regardless of the hormone receptor status), we usually suggest single agent HER2-directed therapy. An alternative option would be to add a HER2-directed agent to systemic therapy, provided the patients are aware of the additional risks associated with combined treatment.

For medically frail patients who are not candidates for surgery, we do not recommend primary radiation therapy unless for the control of local symptoms.

Medically frail women with a limited life expectancy and those who wish to avoid treatment and the associated risk of toxicity should be offered supportive care and referral for palliative care services.

There is a critical need for further research of the appropriate management of elderly breast cancer patients. Clinical trials are required, combined with a change in attitude regarding the suitability of elderly patients for clinical trial enrollment. Of particular importance is the assessment of adjuvant treatment in frail patients who make up the majority of elderly women with breast cancer.

Closer collaboration between oncologists and geriatricians is an imperative. In addition to the development of clinical screening tools for aging, the potential for alternative measures of aging should be also considered. The search for biomarkers of aging has been an area of growing interest in the field of geriatric biology. Extrapolation of aging biomarkers or metabolomics into oncology remains theoretical at present but would potentially provide an important tool for the identification of patients at risk of the toxicities of the adjuvant chemotherapy.
